# Continuous locomotor activity monitoring to assess animal welfare following intracranial surgery in mice

**DOI:** 10.3389/fnbeh.2024.1457894

**Published:** 2024-09-04

**Authors:** Mazyar Abdollahi Nejat, Oliver Stiedl, August B. Smit, Ronald E. van Kesteren

**Affiliations:** Department of Molecular and Cellular Neurobiology, Center for Neurogenomics and Cognitive Research, Amsterdam Neuroscience, Vrije Universiteit Amsterdam, Amsterdam, Netherlands

**Keywords:** animal welfare, locomotor activity, circadian rhythm, stereotaxic surgery, intracranial surgery, home cage monitoring, Alzheimer’s disease

## Abstract

Locomotor activity can serve as a readout to identify discomfort and pain. Therefore, monitoring locomotor activity following interventions that induce potential discomfort may serve as a reliable method for evaluating animal health, complementing conventional methods such as body weight measurement. In this study, we used the digital ventilated cage (DVC^®^) system for the assessment of circadian locomotor activity, in addition to body weight monitoring, following intracranial stereotaxic surgery in an Alzheimer’s disease mouse model (C57BL/6J/APPswe/PSEN1dE9). Stereotaxic surgery did not affect the organization of circadian locomotor activity of both 7–8-week-old and 19–21-week-old mice. However, we observed that both young and old mice exhibited a significant decrease in activity during the dark phase. Also, our study shows that changes in locomotor activity exhibit higher sensitivity in detecting alterations indicative of animal health compared to measuring body weight. In contrast to 7–8-week-old mice, where we observed no genotypic differences in locomotor activity, 19–21-week-old APP/PS1 mice showed increased locomotor activity compared to wild-type mice. Furthermore, our analyses revealed that a subset of the 7–8-week-old mice showed increased locomotor activity during the initial peak of the dark phase. One mouse experienced sudden death early in life, possibly due to epileptic seizures. Altogether, our findings affirm continuous activity measurements as used in the DVC^®^ as a highly valuable objective method for post-surgical welfare monitoring. Its discerning capacity not only facilitates circadian locomotor rhythm assessment but also enables the identification of individual aberrant activity patterns, possibly indicative of epileptic seizures.

## 1 Introduction

Stereotaxic neurosurgery is commonly used in rodent neuroscience research. It enables precise access to specific brain regions, allowing researchers to establish causal relationships between molecular, cellular and physiological processes in the brain and the behavior of the animal. The expanding body of knowledge regarding optimizing surgical methodologies in rodents (Ferry and Gervasoni, 2021; Richardson and Flecknell, 2005; Stokes et al., 2009), alongside compliance with the EU directive (Directive 2010/63/EU, 2010) has fostered the refinement of stereotaxic surgical procedures and the development of standardized protocols for post-surgical care. However, despite these advancements, stereotaxic surgeries could potentially lead to discomfort induced by postoperative pain and affect several physiological parameters (Wassermann et al., 2020). Therefore, to not only ensure the ethical treatment of the animals but also the reliability of experimental outcomes, it is imperative to maintain vigilant post-surgery welfare monitoring.

For mice, current methods that are employed to assess health include observing behavior, assessing physical appearance, as well as measuring body weight. Long-standing evidence has established that particularly body weight can serve as an indicator of animal distress with the advantage that it can be objectively measured (Morton and Griffiths, 1985; Redgate et al., 1991). Body weight measurements have also been used to define humane endpoints. A commonly suggested criterion to apply humane endpoints, to prevent unnecessary suffering, is a 20% reduction of body weight compared to baseline body weight (Morton, 2000; Olfert, 1996). Nevertheless, changes in body weight involve intricate physiological regulatory processes and can be influenced by various factors such as metabolic alterations, increased energy expenditure, and reduced appetite. Hence, it is strongly suggested to consider experiment- and model-specific factors when assessing weight reduction and to complement body weight assessment with additional criteria to evaluate discomfort levels and assess the overall health status of rodents (Talbot et al., 2020).

Monitoring locomotor activity has been shown to be a valuable approach for assessing mouse behavioral patterns (Pernold et al., 2019; Zentrich et al., 2021). Changes in locomotor activity could serve as a valid readout of distress and pain (Whittaker and Howarth, 2014). Specifically, a reduction in locomotor activity has been associated with experiences of pain and distress in prior studies (Alsalem et al., 2020; Jansen Van’t Land and Hendriksen, 1995). Hence, monitoring locomotor activity following interventions that could lead to potential discomfort, such as stereotaxic surgeries, could offer a reliable method for assessing animal welfare. An increasingly used technology that is reliant on the analysis of locomotor behavior, known as home cage monitoring (HCM), has demonstrated its effectiveness in non-intrusively evaluating animal welfare and detecting disease symptoms in mice (Gaburro et al., 2022; Iannello, 2019; Pernold et al., 2019). Moreover, HCM itself is an animal-friendly behavior test environment that eliminates unspecific stressors (Kas et al., 2011; Hager et al., 2014; d'Isa and Gerlai, 2023; d'Isa et al., 2024). Despite the growing number of studies involving HCM, its utilization for the evaluation of post-operative animal welfare remains notably underexplored.

The assessment of locomotor activity is not only used as a viable marker for animal welfare, but changes in locomotor activity and rhythmicity are also indicative of altered circadian clock function and widely adopted as behavioral read-out to study general circadian function in various disease models, including Alzheimer’s disease (AD) (Sheehan and Musiek, 2020). Several studies across diverse AD mouse models reveal alterations in circadian locomotor rhythms in older mice compared to younger mice (Vloeberghs et al., 2004; Wu et al., 2018). Exploring locomotor rhythms in AD mice subjected to surgery could elucidate whether such interventions affect AD mice differently and could potentially offer valuable insight into postoperative recovery processes.

In the present study, we monitored locomotor activity of both APPswe/PSEN1dE9 (APP/PS1 in short) mice and their wild-type (WT) littermates following intracranial stereotaxic surgery using the digital ventilated cage (DVC^®^) system. Disruptions in circadian locomotor rhythm resulting from the surgical procedure were assessed in two distinct age groups: pre-symptomatically, at 7–8 weeks and at 19–21 weeks of age, when cognitive deficits have been reported in APP/PS1 mice (Hijazi et al., 2020; Zhu et al., 2017). In addition to exploring how genotype may influence circadian locomotor rhythm, we examined the efficacy of monitoring locomotor activity as a method for post-surgery health assessment in comparison to body weight measurements. Finally, we discuss the potential use of locomotor activity monitoring to detect animals that are at risk of developing post-operative epileptic seizures.

## 2 Materials and methods

### 2.1 Animals

Experiments were performed on male APP/PS1-SST-cre mice. These mice were generated by crossbreeding APPswe/PSEN1dE9 (The Jackson Laboratory; named APP/PS1) with SST-cre mice (The Jackson Laboratory). APP/PS1 are double transgenic mice that express both the chimeric human/mouse APP gene (Mo/HuAPP695swe) and the mutant human presenilin 1 (PS1) gene with a deletion of exon 9 expressed under the prion protein promotor (MoPrP.Xho) (Jankowsky et al., 2001, 2003, 2004). SST-cre mice are transgenic mice that have an internal ribosomal entry site (IRES) and Cre recombinase in the 3’R UTR of the somatostatin locus, allowing specific expression of a fluorescent protein in somatostatin-positive interneurons using a Cre-dependent viral vector (Taniguchi et al., 2011). All mouse lines were maintained on a C57BL/6JCrl background (Charles River Laboratories). Wild-type SST-cre and transgenic APP/PS1-SST-cre littermate mice at an age of 7–9 weeks or 19–21 weeks were used in this study. Mice were individually housed on a reversed 12-h light/dark cycle with *ad libitum* access to food and water. All experimental procedures were approved by the Central Committee for Animal Experiments (CCD) and the Animal Welfare Body of the Vrije Universiteit Amsterdam in full compliance with the directive 2010/63/EU.

### 2.2 Anesthesia and analgesia

All mice received the analgesic carprofen (0.067 mg/mL; RIMADYL Cattle) in their drinking water 1 day before surgery (Ingrao et al., 2013). Buprenorphine (0.05 mg/kg; Temgesic; RB Pharmaceuticals, United Kingdom) was injected subcutaneously 30–60 min prior to surgery. The mice were initially administered inhalation anesthesia in the isoflurane induction box (~3.5%) for approximately 10 min or until the animal was fully anesthetized. Mice were subsequently positioned on the stereotaxic frame, receiving a continuous supply of isoflurane (~2%, adjusted as necessary) throughout the surgery, which lasted for a duration ranging from 45 to 60 min (Supplementary material). In addition, 50 μL of lidocaine (2%, Sigma-Aldrich, The Netherlands) was administered locally to the skull periosteum to improve analgesia of the periosteum. Carprofen (0.067 mg/mL) was added to the drinking water post-surgery for a minimum of 2 days or continued until body weight either stabilized or returned to its pre-surgery level.

### 2.3 Home cage activity monitoring: digital ventilated cage (DVC^®^) system

The 24/7 activity of mice was continuously monitored in their home cages using the commercial DVC^®^ system (Techniplast, Buguggiate, Italy). The DVC^®^ system uses a capacitance sensing technology, based on a sensing board installed at each cage position that consists of twelve electrodes. This enables detection of differences in the electrical capacitance every 0.25 s (4 Hz) per electrode and thus a total of 48 capacitance measurements per second (Iannello, 2019; Pernold et al., 2019). The placement of animal cages within the DVC^®^-rack was randomized. Cages were exclusively taken out of the rack for surgery, weighing, or at the end of the experiment. Mice were checked for their weight on the day of surgery and the following 3 days. A subset of the animals was weighed daily after the surgery until the end of the experiment.

### 2.4 Data analyses

Data were analyzed using the cloud-based software DVC^®^ Analytics (Techniplast, Buguggiate, Italy). Data were extracted in 1-min bins from the Animal Locomotion Index Smoothed, which is based on activation density (Iannello, 2019). For analysis and presentation, the data was averaged over each hour. For graphical representation the average hourly values were plotted at the center of each respective hour. Prism 10.0.2 (GraphPad Software) was used for visualization and statistical testing. Statistical analysis of data with two genotypes involved the use of two-way repeated measures ANOVA or mixed-effects analysis, along with a Greenhouse–Geisser correction for non-spherical data. For data where genotypes were grouped, one-way (repeated measures) ANOVA was used. When ANOVA or mixed-effects analysis revealed significant differences (*p* < 0.05), Bonferroni’s *post-hoc* multiple comparisons test was applied. The correlation between body weight and average total activity during the dark phase was determined by the Pearson correlation coefficient. Pearson’s *r* was computed for each mouse individually, and the average *r* value was then calculated across all mice per group. In addition, to correct for potentially inflated type I error due to multiple comparisons, we used the false discovery rate (FDR) approach (Benjamini and Hochberg, 1995) to deflate the risk of low scientific replicability (Kafkafi et al., 2018). *p*-values were corrected by the minimum FDR with a threshold of 5%, using a previously reported procedure (Verhoeven et al., 2005). Thereby, the *p*-value to accept statistical significance was *p* < 0.02. Reported values are presented as mean ± standard error of the mean.

## 3 Results

### 3.1 Circadian locomotor rhythm of 7–8-week-old mice

**Figure 1:**
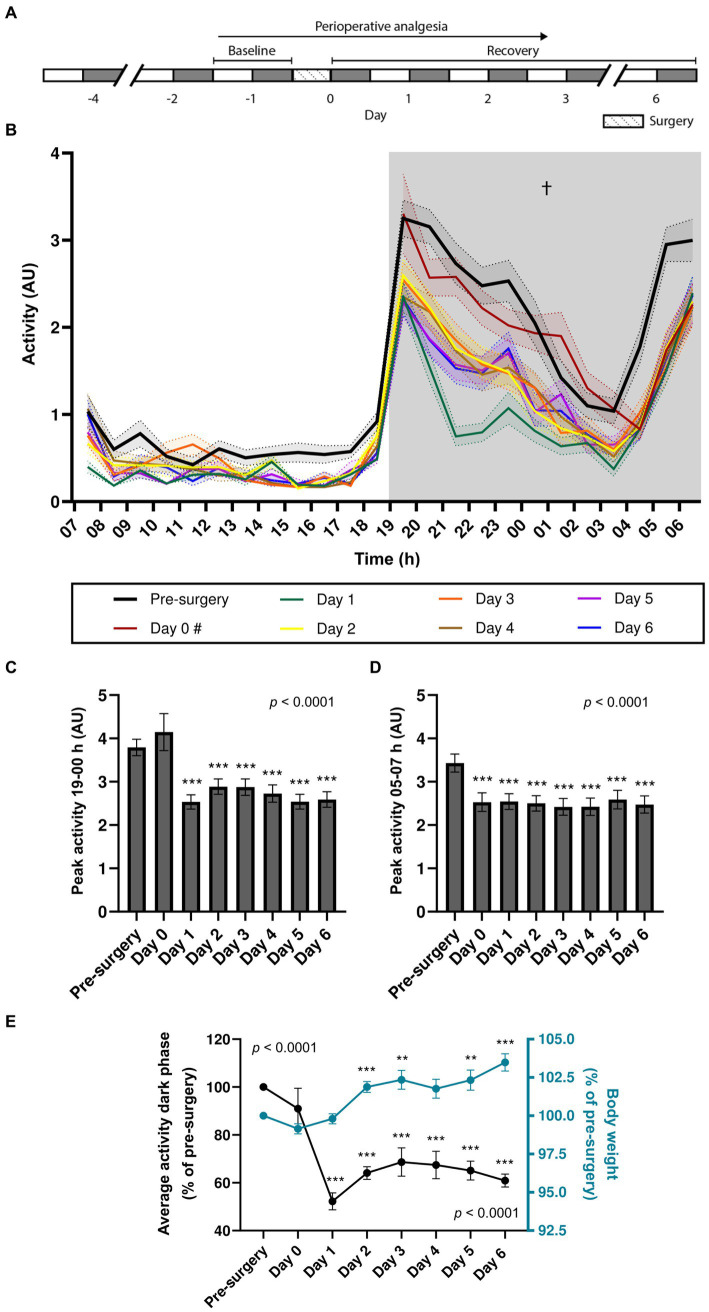
Effects of stereotaxic surgery on the circadian locomotor rhythm of 7–8-week-old mice. **(A)** Experimental timeline (grey: dark phase; white: light phase). **(B)** Activity of WT and TG mice (*n* = 39) during the light and dark phase of the day prior to surgery (pre-surgery), the day of surgery (day 0), and the days after surgery (days 1–6). # Light phase data for day 0 is not available as surgeries were conducted during this period. **(C)** Peak activity between 19 and 00 h. **(D)** Peak activity between 05 and 07 h. **(E)** Average activity during the dark phase (left y-axis; *p* < 0.001 effect of surgery [one-way repeated-measures ANOVA)] in comparison to body weight [right y-axis; *p* < 0.001 effect of surgery (mixed-effects analysis)], both expressed as percentages of pre-surgery levels. † Main effect of surgery; ** *p* < 0.01; *** *p* < 0.001.

We first assessed the effects of stereotaxic surgery on the circadian locomotor rhythm of 7-8-week-old mice. A cohort comprising wild-type (*n* = 13; WT) and transgenic (*n* = 26; TG) APP/PS1 animals was placed in the DVC^®^ system 2–4 days prior to surgery, to allow for habituation after cage-change (Pernold et al., 2019), and their locomotor activity was continuously monitored until 6 days post-surgery (Figure 1A). Two mice, 1 WT and 1 TG, with missing activity data for a duration of at least one full dark phase of the circadian cycle were excluded from analysis and were not included in the sample size reported here. When analyzing total activity during the dark phase, two-way repeated measures ANOVA revealed a main effect of surgery (*p* < 0.0001) but did not show a main effect of genotype or an interaction effect (Table 1). Both pre- and post-surgery, mice exhibited an immediate increase in activity during the transition from the light to dark phase, followed by a decrease in activity over time. Activity started to rise from 04 h, peaking just before the light phase, with only a mildly shifted onset on day 0. Peak activity at both these time points did not show significant differences between genotypes (Supplementary Figures S1A,B). The high level of locomotor activity observed at day 0 in comparison to all other post-surgery days (a level which was comparable to the pre-surgery baseline) and the delayed onset of the second activity peak are likely attributable to the transient effects of the buprenorphine administered during the surgical procedure (Jirkof et al., 2015). Given the absence of significant genotypic effects on activity, data from WT and TG mice were pooled for further analysis. In the pooled data, one-way repeated measures ANOVA showed that activity during the dark phase significantly differed between days [*F*_(3.89, 147.90)_ = 37.67, *p* < 0.0001] (Figure 1B). Except for day 0 (*p* = 0.071), Bonferroni’s *post-hoc* multiple comparisons test showed significant differences between pre-surgery activity and all the post-surgery days (*p* < 0.0001 for pre-surgery vs. day 1–6), indicating that stereotaxic surgery resulted in a long-lasting decrease in activity during the dark phase. Stereotaxic surgery did not affect circadian rhythm organization. One-way repeated measures ANOVA showed an overall effect of surgery on the activity peak amplitude during the first 5 h after the onset of the dark phase between the days [*F*_(1.78, 67.70)_ = 67.70, *p* < 0.0001] (Figure 1C), with Bonferroni’s multiple comparisons test showing differences between pre-surgery and days 1–6 (*p* < 0.0001), indicating that stereotaxic surgery decreased the peak activity amplitude between 19 and 00 h on days 1–6 but not during the dark phase immediately following surgery (day 0). Surgery also significantly affected the peak activity between 05 and 07 h [*F*_(5.13, 195.10)_ = 5.60, *p* < 0.0001], with Bonferroni’s multiple comparisons test showing differences between pre-surgery and all post-surgery days (*p* = 0.0005 for pre-surgery vs. day 0; *p* < 0.0001 for pre-surgery vs. days 1–6, respectively). This indicates a decrease in peak activity amplitude in the hour before the dark-to-light transition on all post-surgery days (Figure 1D). We computed the average total activity during the dark phase for all post-surgery days relative to baseline total activity pre-surgery and observed, in contrast to an increase in body weight, a significant reduction in locomotor activity following stereotaxic surgery (Figure 1E). One-way repeated-measures ANOVA showed an overall effect of surgery on the average activity during the dark phase [*F*_(7, 304)_ = 11.19, *p* < 0.0001], with Bonferroni’s multiple comparisons test showing differences between pre-surgery and days 1–6 (*p* < 0.0001). High variability of day 0 activity is likely due to variations in surgery timing. Remarkably, average activity during the dark phase did not return to baseline levels after 6 days. Mixed-effects analysis indicated a significant effect of stereotaxic surgery on body weight [*F*_(3.14, 83.77)_ = 32.47, *p* < 0.0001], with Bonferroni’s multiple comparisons test revealing significantly higher body weight on days 2–3 (*p* < 0.0001 and *p* = 0.0021, respectively) and days 5–6 (*p* = 0.0046 and *p* < 0.0001, respectively), compared to pre-surgery body weight. In addition, we observed no correlation between body weight and the average activity during the dark phase on post-surgery days (Supplementary Figure S2A). These findings suggest that locomotor activity may be a more sensitive indicator of well-being after surgery than body weight, and that locomotor activity after stereotaxic surgery is independent of body weight changes.

**Table 1:** ANOVA/Mixed-effects model table for dark phase activity of 7–8 and 19–21-week-old mice.

Factor	7–8-week-old mice		19–21-week-old mice	
	*F*_(DFn, DFd)_	*p*-value	*F*_(DFn, DFd)_	*p*-value
Surgery	*F*_(3.889, 143.9)_ = 32.43	*p* < 0.0001	*F*_(1.700, 10.48)_ = 6.356	*p* = 0.0183
Genotype	*F*_(1, 37)_ = 0.8429	*p* = 0.3645	*F*_(1, 7)_ = 14.16	*p* = 0.0071
Surgery x Genotype	*F*_(7, 259)_ = 0.7793	*p* = 0.6052	F_(12, 74)_ = 1.319	*p* = 0.2262

### 3.2 Circadian locomotor rhythm of 19–21-week-old mice

**Figure 2:**
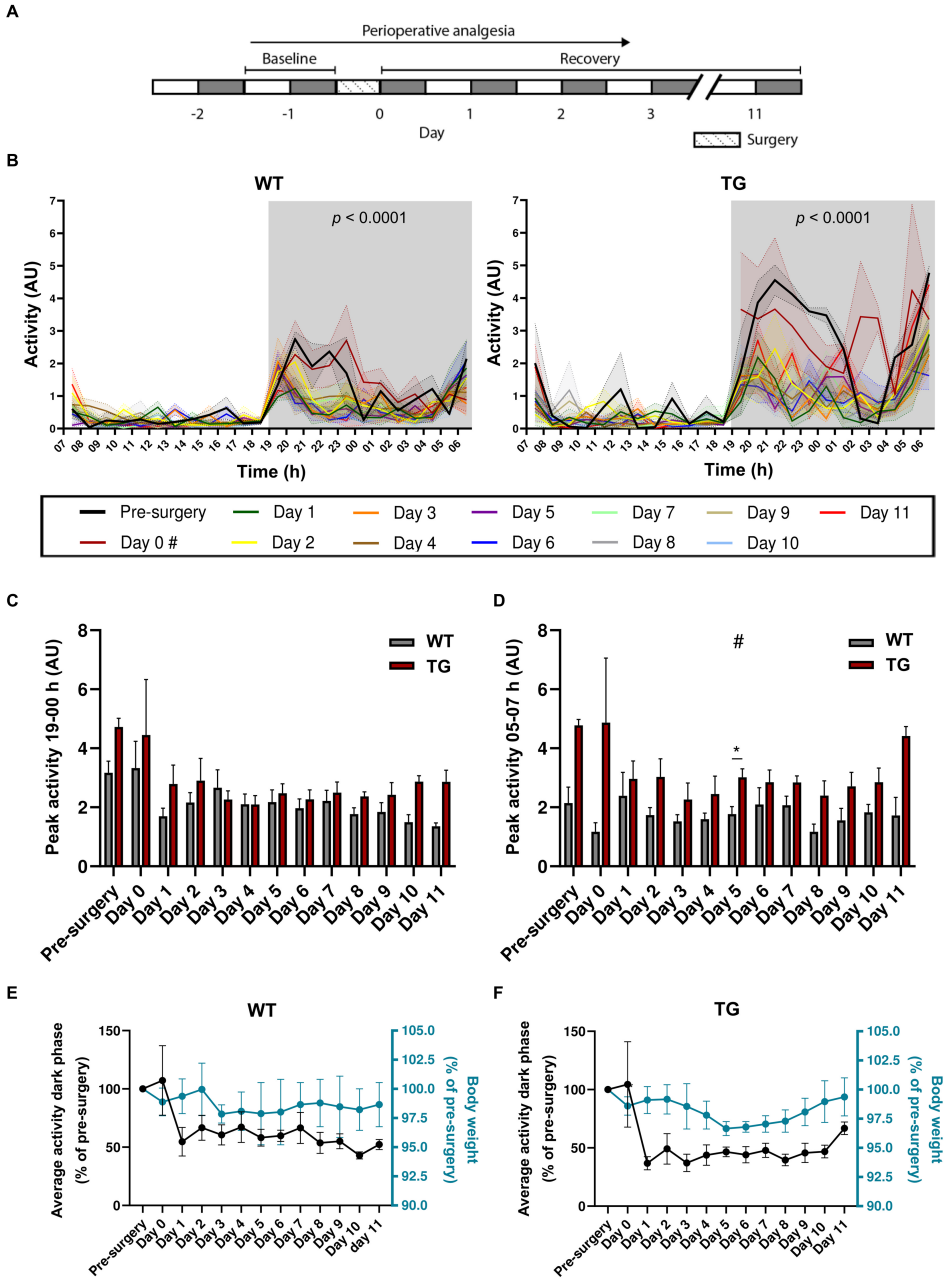
Effects of stereotaxic surgery on the circadian locomotor rhythm of 19–21-week-old mice. **(A)** Experimental timeline (grey: dark phase; white: light phase). **(B)** Activity of WT (left; *n* = 4) and TG (right; *n* = 5) mice during the light and dark phase of the day prior to surgery (pre-surgery), the day of surgery (day 0), and the days after surgery (days 1–11). *p* < 0.001 effect of surgery (mixed-effects analysis). # Light phase data for day 0 is not available as surgeries were conducted during this period. **(C)** Peak activity between 19 and 00 h. **(D)** Peak activity between 05 and 07 h. **(E)** Average activity during the dark phase (left y-axis) in comparison to body weight (right y-axis), both expressed as percentages of pre-surgery levels of WT and **(F)** TG mice. # Main effect of genotype; *p < 0.05.

Next, we aimed to assess the effects of stereotaxic surgery on older animals aged 19–21 weeks. A cohort comprising WT (*n* = 4) and TG (*n* = 5) APP/PS1 mice was recorded for a period of 11 days post-surgery (Figure 2A). Mixed-effects analysis showed a main effect of both surgery and genotype on total activity during the dark phase (Table 1). We observed increased activity in TG mice compared to WT mice, while circadian locomotor rhythm organization was similar between the two groups (Figure 2B). In WT mice, mixed-effects analysis showed a significant effect of surgery on activity [*F*_(2.72, 59.61)_ = 9.83, *p* < 0.0001]. However, Bonferroni’s multiple comparisons test revealed no significant differences in locomotor activity between pre-surgery and all post-surgery days. In TG mice, mixed-effects analysis also showed a significant effect of surgery [*F*_(2.63, 57.89)_ = 15.12, *p* < 0.0001], with Bonferroni’s multiple comparisons test revealing a significant difference in activity between pre-surgery and day 1, 3–5, 7, and 10 (*p* = 0.0083; *p* = 0.017; *p* = 0.0083; *p* = 0.011; *p* = 0.014; *p* = 0.018, respectively). Similar to 7–8-week-old mice, two circadian activity peaks were observed in both genotypes: one at the onset of the dark phase and an anticipatory peak at the end of the dark phase. In addition, a trend towards significantly increased peak activity between 19 and 00 h was observed in TG mice (Figure 2C; Table 2), while mixed-effect analysis revealed a main effect of genotype on activity peak amplitude between 05 and 07 h (Figure 2D; Table 2). Bonferroni’s multiple comparisons test revealed a significant increase in peak activity in TG mice between 05–07 h on day 5 (*p* = 0.015). This increase in activity during the second half of the active phase has been described earlier as sundowning-like behavior in 6-month-old APP23 mice (Vloeberghs et al., 2004). Using mixed-effects analysis, we found that surgery did not significantly affect the average total activity during the dark phase in both WT [*F*_(1.92, 7.35)_ = 2.74, *p* = 0.13] (Figure 2E) and TG mice [*F*_(1.16, 3.08)_ = 5.18, *p* = 0.10] (Figure 2F). Similarly, there was no significant effect on body weight in either WT [*F*_(1.27, 2.23)_ = 0.26, *p* = 0.71] (Figure 2E) and TG mice [*F*_(0.32, 0.47)_ = 1.82, *p* = 0.38] (Figure 2F). Exclusion of day 0 values resulted in significant effects only on activity of TG mice (see Supplementary material). Again, similar to 7–8-week-old mice, we did not observe a correlation between body weight and average total activity during the dark phase (Supplementary Figure S2B). When comparing average total activity during the dark phase between 7–8-week-old and 19–21-week-old mice, we did not observe any statistically significant differences (Supplementary Figure S3A). On the other hand, two-way repeated measures ANOVA indicated a significant main effect of surgery on locomotor activity [*F*_(1.84, 77.42)_ = 10.11, *p* = 0.0002], with Bonferroni’s multiple comparisons test revealing a significant decrease in activity of 7–8-week-old mice on days 1–6 (*p* < 0.0001) and on day 1, 2, 5, and 6 for 19–21-week-old mice (*p* = 0.0477; *p* = 0.0085; *p* = 0.026; *p* < 0.0001, respectively), with no main effect of age and no interaction effect (Supplementary Table S1). This indicates that stereotaxic surgery affects activity of young and old mice similarly. In addition, we used two-way repeated measures ANOVA to compare percentage of change in body weight of 7–8-week-old mice with the change in body weight of 19–21-week-old animals (Supplementary Figure S3B). A main effect of surgery [*F*_(3.95, 127.4)_ = 6.28, *p* = 0.0001], and of age [*F*_(1, 46)_ = 10.06, *p* = 0.0027], as well as an interaction effect [*F*_(7, 266)_ = 5.69, *p* < 0.001] were found (Supplementary Table S2). Post-hoc Bonferroni’s multiple comparisons test showed a statistically significant increase in body weight of 7–8-week-old mice, but not in 19–21-week-old mice, on days 3–7 (*p* < 0.0001; *p* = 0.0021; *p* = 0.017; *p* = 0.0027; *p* < 0.0001, respectively). Between the two age groups, Bonferroni’s multiple comparisons test did not show statistically significant differences on any of the post-surgery days. Overall, these results suggest a significant effect of stereotaxic surgery on locomotor activity of 19–21-week-old mice, while circadian locomotor rhythm organization and body weight remain largely unaffected after surgery. This preliminary finding supports the results observed in 7–8-week-old mice, suggesting that locomotion may be the most sensitive measure of post-surgery animal welfare. However, it is important to note that due to the small sample size (*n* = 4–5 per group) the result for 19–21-week-old mice is preliminary.

**Table 2:** Mixed-effects model table for peak activity dark phase of 19–21-week-old mice.

Factor	Peak activity 19–00 h		Peak activity 05–07 h	
	*F*_(DFn, DFd)_	*p*-value	*F*_(DFn, DFd)_	*p*-value
Surgery	*F*_(2.326, 14.34)_ = 2.807	*p* = 0.0876	*F*_(2.326, 14.34)_ = 1.426	*p* = 0.2745
Genotype	*F*_(1, 7)_ = 5.286	*p* = 0.0551	*F*_(1, 7)_ = 13.81	*p* = 0.0075
Surgery x Genotype	*F*_(12, 74)_ = 0.7094	*p* = 0.7376	*F*_(12, 74)_ = 1.353	*p* = 0.2085

### 3.3 Increased peak activity: a potential indicator of epileptic seizures?

**Figure 3:**
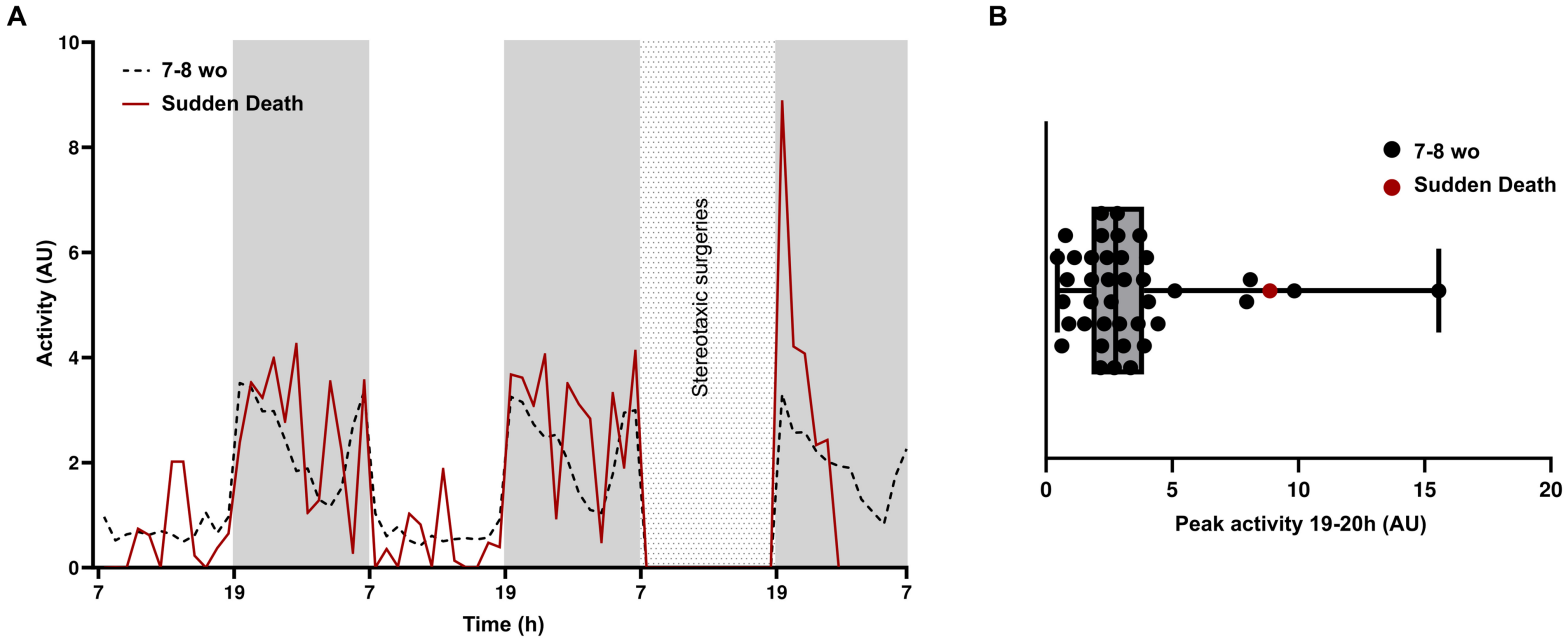
Post-surgery hyperactivity in a mouse with early sudden death. **(A)** Average activity of 7–8-week-old mice (*n* = 39) and average activity of the mouse that experienced early sudden death, during the light and dark phases of 2 days prior to surgery and during the dark phase immediately after surgery. **(B)** Box plot showing the peak amplitude of activity between 19 and 22 h.

Within the group of 7–8-week-old animals, one APP/PS1 mouse experienced sudden death. Considering the mouse’s rigid posture and extended legs when finding it in the cage (Milh et al., 2020), alongside documented instances of spontaneous seizures and epileptiform activity in this mouse line (Minkeviciene et al., 2009), we conclude that the sudden death was likely due to an epileptic seizure. We compared the activity of this mouse with the average activity of all other 7–8-week-old animals (Figure 3A). Activity levels during the 2 days prior to the surgical procedure were not different. However, at the onset of the dark phase following surgery, between 19 and 20 h, the mouse that died exhibited hyperactivity. This surge of activity coincided with the initial peak observed in other animals and occurred a few hours preceding the sudden death. When plotting individual peak activities of all mice, we observed four other mice with peak activities at more than 1SD higher than the mean (Figure 3B). Based on this observation, we hypothesize that high activity levels during the initial peak of the dark phase may be indicative of increased epileptic activity that may result in sudden death. Future electroencephalography (EEG) or electrocorticography (ECoG) investigations (Kovacevic et al., 2018) are needed to ascertain a definitive correlation between this increased peak activity and epileptic events.

## 4 Discussion

Although locomotor activity has been utilized to examine circadian rhythms in mice (Hughes, 2018), there is little research exploring the impact of surgical procedures on circadian locomotor behavior. While previous studies have focused on non-intracranial procedures (Leon et al., 2004), have examined locomotor activity post-surgery in rats (Wassermann et al., 2020), or examined locomotor activity after brain injuries or lesions (Shimizu and Fukada, 2017; Qu et al., 2016), this study notably represents the first 24/7 assessment of locomotor activity of mice in their home cage for ~1–2 weeks from shortly before and after intracranial surgery. In the current study, we assessed the effects of stereotaxic surgery, a commonly used method in rodent neuroscience research, on the circadian locomotor behavior of mice using the commercially available digital ventilated cage (DVC^®^) system. We used both C57BL/6 J/APPswe/PSEN1dE9 and WT (C57BL/6 J) mice, including a comparison between the two, to ensure that our findings are not limited to the specific transgenic strain but are broadly applicable to the commonly used C57BL/6 J background. Since the mice used in this study were sourced from a group allocated for a subsequent study that focused exclusively on males, we were unable to include female mice in the current study. However, previous studies have shown no locomotor differences between male and female mice following surgical procedures (Warner and Padmanabhan, 2020). In addition, necessitated by the design of that subsequent study, in which two groups of TG and one group of WT mice were needed, we used unequal group sizes in the experiments with 7–8-week-old mice. All mice used in this study underwent surgery to deliver adeno-associated virus (AAV) (Lowery and Majewska, 2010), to chemogenetically manipulate the activity of certain types of interneurons in the hippocampus. The primary objective was to monitor circadian locomotor behavior post-surgery without any additional manipulative interventions. The viral vectors used were for fluorescent labelling and/or DREADD expression (see Supplementary material). While targeting certain genes with viral vectors could lead to varying post-surgery effects, depending on the turnover of the target genes and their biological impact, we expected no negative effects from the expression of the viral vectors used in this study. The DVC^®^ system facilitates the recording of locomotor activity through electromagnetic field technology (EMF), allowing for continuous monitoring of mice within their home cage (Iannello, 2019). The suitability of this system for HCM provides a controlled environment that enhances the accuracy of our observations by minimizing external influences and ensuring consistent monitoring conditions (Fuochi et al., 2021; Golini et al., 2023). The exposure to EMF does not induce any short- or long-term biological effects, nor does it affect cognition or overall welfare (Burman et al., 2018; Recordati et al., 2019).

We identified two peaks in activity: one occurring shortly after the lights were turned off and another anticipatory peak just before the end of the dark phase, both of which have been reported before for C57BL/6 J mice using two different home cage monitoring systems (Hager et al., 2014; Loos et al., 2014). Stereotaxic surgery resulted in a significant decrease in activity in 7–8-week-old mice, consistent with the preliminary findings in 19–21-week-old mice, while the overall organization of the circadian rhythm, i.e., the presence of two activity peaks during the dark phase, remained unaltered. Our observations are consistent with earlier rodent studies, which indicated a significant decline in activity following surgical interventions (Leon et al., 2004; Wassermann et al., 2020). The observed effects are not likely attributable to the inhalation anesthetic isoflurane, since prior research has demonstrated that comparable quantities and durations of inhalation anesthesia did not impact locomotor activity (Cesarovic et al., 2010). While performing procedures during the light cycle may disrupt the rodent’s resting period (Hawkins and Golledge, 2018), earlier studies have established that circadian rhythms of locomotor activity are less sensitive to isoflurane anesthesia when administered during the rest phase compared to when administered during the dark phase (Kikuchi et al., 2013; Poulsen et al., 2018). In addition, in both 7–8-week-old mice and 19–21-week-old mice, we did not observe changes in body weight during the first 2 days after stereotaxic surgery. However, in 7–8-week-old mice body weight significantly increased from day 3 onwards. This could possibly be attributable to the normative weight gain trajectory observed in younger mice, which occurs at a higher rate than observed in older mice (Lin et al., 2019; Rendina-Ruedy et al., 2015). During this entire period, locomotor activity was significantly decreased after stereotaxic surgery and did not revert to baseline levels, implying that locomotor activity exhibits higher sensitivity to detect post-surgery discomfort compared to body weight change, as observed in earlier studies (Gaburro et al., 2022). Furthermore, our results suggest increased activity in 19–21-week-old APP/PS1 mice compared to WT mice, both at baseline and post-surgery, whereas this was not observed in 7–8-week-old mice. Previous studies investigating the effect of genotype on locomotor activity in AD mouse models have yielded variable results. Whereas our results align with previous studies reporting increased activity during the second half of the active phase in 6-month-old APP23 mice, reminiscent of the sundowning phenomenon observed in Alzheimer patients (Vloeberghs et al., 2004), other studies have reported no differences in locomotor activity between WT and APP/PS1 male mice aged between 3.5 and 5.5 months (Baño Otalora et al., 2012). In addition, altered levels and patterns in circadian locomotor activity are reported in APP/PS1 mice of 2 months (Oyegbami et al., 2017), an age comparable to the one of the mice used in our study. However, it is crucial to underscore that these earlier reported variations apply to female mice, whereas our study exclusively utilized male mice.

Similar to AD patients, who face an increased risk of developing epilepsy (Yang et al., 2022), many transgenic AD animal models exhibit abnormal electrical activity (Reyes-Marin and Nuñez, 2017). Animals carrying the Swedish APP mutation, such as APP23 and APP/PS1dE9, have frequently been reported to have spontaneous seizures and epileptiform activity (Lalonde et al., 2005; Minkeviciene et al., 2009). In addition, a 5–20% age-dependent mortality rate in APP/PS1 mice on a C57BL/6 background has been reported due to sudden death early in life (Minkeviciene et al., 2009; Tzeng et al., 2018). This early premature death has been suggested to result from prolonged seizures (Minkeviciene et al., 2009). However, there is a notable lack of studies that have systematically observed epileptic seizures preceding sudden death in AD mouse models. In our study, we observed 5 animals with increased locomotor activity during the dark phase following stereotaxic surgery. In one of these occurrences, this increased locomotor activity preceded premature sudden death. Hence, together with the discovery of the mouse exhibiting a posture indicative of seizures within its cage (Milh et al., 2020), we hypothesize that this surge in activity could be associated with epileptic seizures and increases the risk of premature sudden death (Minkeviciene et al., 2009) and could be an indicator for mice that are at risk of developing such seizures. However, it is imperative to approach the interpretation of these findings with care, given the current absence of direct measurements of seizure activity in the brain.

Despite the valuable insights gained from our investigation, we consider the following limitations. Firstly, our study reveals a significant decrease in average activity during the dark phase, which, notably, did not show recovery to baseline levels by post-surgery day 6 in 7–8-week-old mice. Similar effects induced by stereotaxic surgery were observed in 19–21-week-old mice, although recorded for an extended period, with average activity not restoring to baseline levels. It is known that the recovery of the physical activity depends on the severity of the surgical procedure. The full recovery of baseline activity after radio-transmitter implantation compared to naïve mice for novelty exploration in fear conditioning ranges between 14 and 21 days, so up to 3 weeks are required before locomotor activity to novelty exposure does not differ from that of naïve mice (Stiedl et al., 1999, 2004). However, the duration of recovery of the amplitude of activity to baseline levels after cerebral injection surgery as welfare index has not been reported to date. Therefore, future experiments require an extended recording duration to determine the complete time required for full recovery of locomotor activity after stereotaxic surgery. We are convinced that our subsequent experiments with these mice have been conducted after full recovery, as they started 3–4 weeks after surgery. HCM was restricted to approximately the first week after stereotaxic surgery due to transfer of these mice to another animal facility.

Secondly, in 7–8-week-old mice, we observed a high variability in activity during the initial peak of the dark phase immediately following stereotaxic surgery, but not during the anticipatory peak at the end of the dark phase. This is likely attributable to the interplay between buprenorphine and the varying timing of surgeries among mice (Jirkof et al., 2015). The impact of carprofen in our study remains however unclear. Hence, we suggest future experiments exploring the effects of these analgesia with varying concentrations and/or durations of administration on the activity of mice during the recovery from stereotaxic surgery. This approach would also allow to assess the optimal analgesic dose and duration as further refinement of post-surgery care. The high variability in 19–21-week-old mice is possibly attributed to the relatively limited sample size. In addition, this variability may be explained by a subset of mice experiencing epileptic seizures, likely more prevalent compared to 7–8-week old mice, as a correlation between the amount of amyloid beta plaques and seizure frequency has been previously described (Reyes-Marin and Nuñez, 2017).

Finally, while previous studies have frequently reported spontaneous seizures and epileptiform activity in AD mice (Lalonde et al., 2005; Minkeviciene et al., 2009), and EEG recordings confirm increased epileptiform activity in our APP/PS1 mouse strain as well (unpublished data), our current study lacks direct observational data to support this claim. Therefore, additional experiments are necessary to confirm whether the observed increased activity in the APP/PS1 mouse that experienced premature sudden death is indicative of epileptic seizures and has the potential to serve as a potential indicator for sudden premature death. To achieve this, an effective strategy may include the integration of techniques for recording epileptic seizures, such as video-EEG recordings (Rensing et al., 2012), with HCM, and the identification of events that may predict its occurrence. Nevertheless, circadian activity monitoring is useful for indirect assessment of potential discomfort in novel genetically modified mouse lines of diseases in animal research as animal welfare requirement within the EU (Zintzsch et al., 2017). This may include refined application of humane endpoints based on predictive indicators of symptom progression in disease models such as hypo- and hyperactivity.

In conclusion, we found that stereotaxic surgery significantly decreased locomotor activity in 7–8-week-old mice and observed similar preliminary effects in 19–21-week-old mice, while circadian locomotor rhythm organization remained unaffected. We propose future experiments involving extended recording periods and manipulation of analgesia to assess the impact of stereotaxic surgery on locomotor activity in more detail. Although the duration of our study was insufficient to observe full recovery of locomotor activity to baseline levels, our study emphasizes the importance of an adequate duration of post-surgery recovery before conducting critical experiments and notably highlights higher sensitivity of locomotor activity compared to body weight change. In addition, studies with larger sample sizes at an older age, including 19–21-week-old mice, are required to further validate these findings and enhance the robustness of the conclusions. We expect that our findings apply not only to APP/PS1 and C57BL/6 wildtype mice, but also to other disease models, many of which are associated with progressively increasing discomfort and lethality. We therefore recommend monitoring of locomotor activity in addition to body weight following surgical procedures and/or disease progression. Automated monitoring of locomotor activity can provide valuable insights of the health status and disease progression in experimental animals from an animal welfare perspective. Standardizing such measurements in surgical protocols would be beneficial for assessing (cumulative) discomfort levels, and reporting any potential discomfort as identified by HCM would contribute to the transparency and replicability of studies in line with the ARRIVE 2.0 guidelines (Du Sert et al., 2020).

## Data Availability

The original contributions presented in the study are included in the article/Supplementary material, further inquiries can be directed to the corresponding authors.

## References

[ref1] Alsalem M. Haddad M. Altarifi A. Aldossary S. A. Kalbouneh H. Abojaradeh A. M.. (2020). Impairment in locomotor activity as an objective measure of pain and analgesia in a rat model of osteoarthritis. Exp. Ther. Med. 20:165. doi: 10.3892/etm.2020.9294, PMID: 33093903 PMC7571323

[ref2] Baño Otalora B. B. Popovic N. Gambini J. Popovic M. Viña J. Bonet-Costa V.. (2012). Circadian system functionality, hippocampal oxidative stress, and spatial memory in the APPswe/PS1dE9 transgenic model of Alzheimer disease: effects of melatonin or ramelteon. Chronobiol. Int. 29, 822–834. doi: 10.3109/07420528.2012.699119 22823866

[ref3] Benjamini Y. Hochberg Y. (1995). Controlling the false discovery rate: a practical and powerful approach to multiple testing. J. R Stat. Soc. Series B 57, 289–300. doi: 10.1111/j.2517-6161.1995.tb02031.x

[ref4] Burman O. Marsella G. Clemente A. Cervo L. (2018). The effect of exposure to low frequency electromagnetic fields (EMF) as an integral part of the housing system on anxiety-related behaviour, cognition and welfare in two strains of laboratory mouse. PLoS One 13:e0197054. doi: 10.1371/journal.pone.0197054, PMID: 29771983 PMC5957419

[ref5] Cesarovic N. Nicholls F. Rettich A. Kronen P. Hässig M. Jirkof P.. (2010). Isoflurane and sevoflurane provide equally effective anaesthesia in laboratory mice. Lab. Anim. 44, 329–336. doi: 10.1258/la.2010.009085, PMID: 20507878

[ref6] Directive 2010/63/EU (2010). Directive 2010/63/EU of the European Parliament and of the Council of 22 September 2010 on the protection of animals used for scientific purposes. Off. J. Eur. Union 276, 33–79.

[ref7] d'Isa R. Fasano S. Brambilla R. (2024). Editorial: animal-friendly methods for rodent behavioral testing in neuroscience research. Front. Behav. Neurosci. 18:1431310. doi: 10.3389/fnbeh.2024.1431310, PMID: 38983871 PMC11232432

[ref8] d'Isa R. Gerlai R. (2023). Designing animal-friendly behavioral tests for neuroscience research: the importance of an ethological approach. Front. Behav. Neurosci. 16:1090248. doi: 10.3389/fnbeh.2022.1090248, PMID: 36703720 PMC9871504

[ref9] Du Sert N. P. Hurst V. Ahluwalia A. Alam S. Avey M. T. Baker M.. (2020). The ARRIVE guidelines 2.0: updated guidelines for reporting animal research. PLoS Biol. 18:e3000410. doi: 10.1371/journal.pbio.3000410, PMID: 32663219 PMC7360023

[ref10] Ferry B. Gervasoni D. (2021). Improving stereotaxic neurosurgery techniques and procedures greatly reduces the number of rats used per experimental group-a practice report. Animals 11:2662. doi: 10.3390/ani11092662, PMID: 34573633 PMC8465152

[ref11] Fuochi S. Rigamonti M. Iannello F. Raspa M. Scavizzi F. de Girolamo P.. (2021). Phenotyping spontaneous locomotor activity in inbred and outbred mouse strains by using digital ventilated cages. Lab. Animal 50, 215–223. doi: 10.1038/s41684-021-00793-0, PMID: 34155410

[ref12] Gaburro S. Winter Y. Loos M. Kim J. J. Stiedl O. (2022). Editorial: home cage-based phenotyping in rodents: innovation, standardization, reproducibility and translational improvement. Front. Neurosci. 16:894193. doi: 10.3389/fnins.2022.894193, PMID: 35812217 PMC9261870

[ref13] Golini E. Rigamonti M. Raspa M. Scavizzi F. Falcone G. Gourdon G.. (2023). Excessive rest time during active phase is reliably detected in a mouse model of myotonic dystrophy type 1 using home cage monitoring. Front. Behav. Neurosci. 17:1130055. doi: 10.3389/fnbeh.2023.1130055, PMID: 36935893 PMC10017452

[ref14] Hager T. Jansen R. F. Pieneman A. W. Manivannan S. N. Golani I. van der Sluis S.. (2014). Display of individuality in avoidance behavior and risk assessment of inbred mice. Front. Behav. Neurosci. 8:314. doi: 10.3389/fnbeh.2014.00314 25278853 PMC4165351

[ref15] Hawkins P. Golledge H. D. R. (2018). The 9 to 5 rodent − time for change? Scientific and animal welfare implications of circadian and light effects on laboratory mice and rats. J. Neurosci. Methods 300, 20–25. doi: 10.1016/j.jneumeth.2017.05.014, PMID: 28502554

[ref16] Hijazi S. Heistek T. S. Scheltens P. Neumann U. Derya Shimshek R. Van Kesteren R. E. (2020). Early restoration of parvalbumin interneuron activity prevents memory loss and network hyperexcitability in a mouse model of Alzheimers disease. Mol. Psychiatry 25, 3380–3398. doi: 10.1038/s41380-019-0483-4, PMID: 31431685 PMC7714697

[ref17] Hughes A. T. L. (2018). Locomotor exercise and circadian rhythms in mammals. Curr. Opin. Physio. 5, 51–57. doi: 10.1016/j.cophys.2018.07.001

[ref18] Iannello F. (2019). Non-intrusive high throughput automated data collection from the home cage. Heliyon 5:e01454. doi: 10.1016/j.heliyon.2019.e01454, PMID: 30997429 PMC6451168

[ref19] Ingrao J. C. Johnson R. Tor E. Gu Y. Litman M. Turner P. V. (2013). Aqueous stability and oral pharmacokinetics of meloxicam and carprofen in male C57BL/6 mice. J. Am. Assoc. Lab. Anim. Sci. 52, 553–559. 24041210 PMC3784660

[ref20] Jankowsky J. L. Fadale D. J. Anderson J. Xu G. M. Gonzales V. Jenkins N. A.. (2004). Mutant presenilins specifically elevate the levels of the 42 residue beta-amyloid peptide in vivo: evidence for augmentation of a 42-specific gamma secretase. Hum. Mol. Genet. 13, 159–170. doi: 10.1093/hmg/ddh019, PMID: 14645205

[ref21] Jankowsky J. L. Slunt H. H. Ratovitski T. Jenkins N. A. Copeland N. G. Borchelt D. R. (2001). Co-expression of multiple transgenes in mouse CNS: a comparison of strategies. Biomol. Eng. 17, 157–165. doi: 10.1016/s1389-0344(01)00067-3, PMID: 11337275

[ref22] Jankowsky J. L. Xu G. Fromholt D. Gonzales V. Borchelt D. R. (2003). Environmental enrichment exacerbates amyloid plaque formation in a transgenic mouse model of Alzheimer disease. J. Neuropathol. Exp. Neurol. 62, 1220–1227. doi: 10.1093/jnen/62.12.1220, PMID: 14692698

[ref23] Jansen Van’t Land C. Hendriksen C. F. (1995). Change in locomotor activity pattern in mice: a model for recognition of distress? Lab. Anim. 29, 286–293. doi: 10.1258/002367795781088207 7564213

[ref24] Jirkof P. Tourvieille A. Cinelli P. Arras M. (2015). Buprenorphine for pain relief in mice: repeated injections vs sustained-release depot formulation. Lab. Anim. 49, 177–187. doi: 10.1177/0023677214562849 25488320

[ref25] Kafkafi N. Agassi J. Chesler E. J. Crabbe J. C. Crusio W. E. Eilam D.. (2018). Reproducibility and replicability of rodent phenotyping in preclinical studies. Neurosci. Biobehav. Rev. 87, 218–232. doi: 10.1016/j.neubiorev.2018.01.003, PMID: 29357292 PMC6071910

[ref26] Kas M. J. Golani I. Benjamini Y. Fonio E. Stiedl O. (2011). Longitudinal assessment of deliberate mouse behavior in the home cage and attached environments: relevance to anxiety and mood disorders. Mood and anxiety related phenotypes in mice: characterization using behavioral tests, Gould T. Humana Press Totowa, NJ

[ref27] Kikuchi T. Tan H. Mihara T. Uchimoto K. Mitsushima D. Takase K.. (2013). Effects of volatile anesthetics on the circadian rhythms of rat hippocampal acetylcholine release and locomotor activity. Neuroscience 237, 151–160. doi: 10.1016/j.neuroscience.2013.01.062, PMID: 23396087

[ref28] Kovacevic J. Maroteaux G. Schut D. Loos M. Dubey M. Pitsch J.. (2018). Protein instability, haploinsufficiency, and cortical hyper-excitability underlie STXBP1 encephalopathy. Brain 141, 1350–1374. doi: 10.1093/brain/awy046, PMID: 29538625 PMC5917748

[ref29] Lalonde R. Dumont M. Staufenbiel M. Strazielle C. (2005). Neurobehavioral characterization of APP23 transgenic mice with the SHIRPA primary screen. Behav. Brain Res. 157, 91–98. doi: 10.1016/j.bbr.2004.06.020, PMID: 15617775

[ref30] Leon L. R. Walker L. D. DuBose D. A. Stephenson L. A. Stephenson L. A. (2004). Biotelemetry transmitter implantation in rodents: impact on growth and circadian rhythms. Am. J. Physiol. 286, R967–R974. doi: 10.1152/ajpregu.00380.2003, PMID: 14726427

[ref31] Lin Y. S. Lin F. Y. Hsiao Y. H. (2019). Myostatin is associated with cognitive decline in an animal model of Alzheimer’s disease. Mol. Neurobiol. 56, 1984–1991. doi: 10.1007/s12035-018-1201-y, PMID: 29982981

[ref32] Loos M. Koopmans B. Aarts E. Maroteaux G. van der Sluis S. Neuro-BSIK Mouse Phenomics Consortium. (2014). Sheltering behavior and locomotor activity in 11 genetically diverse common inbred mouse strains using home-cage monitoring. PLoS One 9:e108563. doi: 10.1371/journal.pone.0108563, PMID: 25264768 PMC4180925

[ref33] Lowery R. L. Majewska A. K. (2010). Intracranial injection of adeno-associated viral vectors. J. Vis. Exp. 45:2140. doi: 10.3791/2140, PMID: 21113119 PMC3159586

[ref34] Milh M. Roubertoux P. Biba N. Chavany J. Spiga Ghata A. Fulachier C.. (2020). A knock-in mouse model for KCNQ2-related epileptic encephalopathy displays spontaneous generalized seizures and cognitive impairment. Epilepsia 61, 868–878. doi: 10.1111/epi.16494, PMID: 32239694 PMC7317210

[ref35] Minkeviciene R. Rheims S. Dobszay M. B. Zilberter M. Hartikainen J. Fülöp L.. (2009). Amyloid beta-induced neuronal hyperexcitability triggers progressive epilepsy. J. Neurosci. 29, 3453–3462. doi: 10.1523/jneurosci.5215-08.2009, PMID: 19295151 PMC6665248

[ref36] Morton D. B. (2000). A systematic approach for establishing humane endpoints. ILAR J. 41, 80–86. doi: 10.1093/ilar.41.2.80, PMID: 11406701

[ref37] Morton D. B. Griffiths P. H. (1985). Guidelines on the recognition of pain, distress and discomfort in experimental animals and an hypothesis for assessment. Vet. Rec. 116, 431–436. doi: 10.1136/vr.116.16.431, PMID: 3923690

[ref38] Olfert E. D. (1996). Considerations for defining an acceptable endpoint in toxicological experiments. Lab. Anim. 25, 38–43.

[ref39] Oyegbami O. Collins H. M. Pardon M.-C. Ebling F. J. P. Heery D. M. Moran P. M. (2017). Abnormal clock gene expression and locomotor activity rhythms in two month-old female APPSwe/PS1dE9 mice. Curr. Alzheimer Res. 14, 850–860. doi: 10.2174/1567205014666170317113159, PMID: 28317486

[ref40] Pernold K. Iannello F. Low B. E. Rigamonti M. Rosati G. Scavizzi F.. (2019). Towards large scale automated cage monitoring – diurnal rhythm and impact of interventions on in-cage activity of C57BL/6J mice recorded 24/7 with a non-disrupting capacitive-based technique. PLoS One 14:e0211063. doi: 10.1371/journal.pone.0211063, PMID: 30716111 PMC6361443

[ref41] Poulsen R. C. Warman G. R. Sleigh J. Ludin N. M. Cheeseman J. F. (2018). How does general anaesthesia affect the circadian clock? Sleep Med. Rev. 37, 35–44. doi: 10.1016/j.smrv.2016.12.002, PMID: 28162920

[ref42] Qu W. Liu N. K. Xie X. M. Li R. Xu X. M. (2016). Automated monitoring of early neurobehavioral changes in mice following traumatic brain injury. Neural Regen. Res. 11, 248–256. doi: 10.4103/1673-5374.177732, PMID: 27073377 PMC4810988

[ref43] Recordati C. De Maglie M. Marsella G. Milite G. Rigamonti A. Paltrinieri S.. (2019). Long-term study on the effects of housing C57BL/6NCrl mice in cages equipped with wireless technology generating extremely low-intensity electromagnetic fields. Toxicol. Pathol. 47, 598–611. doi: 10.1177/0192623319852353 31117895

[ref44] Redgate E. S. Deutsch M. Boggs S. S. (1991). Time of death of CNS tumor-bearing rats can be reliably predicted by body weight-loss patterns. Lab. Anim. Sci. 41, 269–273, PMID: 1658469

[ref45] Rendina-Ruedy E. Hembree K. D. Sasaki A. Davis M. R. Lightfoot S. A. Clarke S. L.. (2015). A comparative study of the metabolic and skeletal response of C57BL/6J and C57BL/6N mice in a diet-induced model of type 2 diabetes. J. Nutr. Metab. 2015, 1–13. doi: 10.1155/2015/758080, PMID: 26146567 PMC4469802

[ref46] Rensing N. R. Guo D. Wong M. (2012). Video-EEG monitoring methods for characterizing rodent models of tuberous sclerosis and epilepsy. Methods Mol. Biol. 821, 373–391. doi: 10.1007/978-1-61779-430-8_24, PMID: 22125079

[ref47] Reyes-Marin K. E. Nuñez A. (2017). Seizure susceptibility in the APP/PS1 mouse model of Alzheimer’s disease and relationship with amyloid β plaques. Brain Res. 1677, 93–100. doi: 10.1016/j.brainres.2017.09.026 28963050

[ref48] Richardson C. A. Flecknell P. A. (2005). Anaesthesia and post-operative analgesia following experimental surgery in laboratory rodents: are we making progress? Alternat. Lab. Anim. 33, 119–127. doi: 10.1177/026119290503300207, PMID: 16180987

[ref49] Sheehan P. W. Musiek E. S. (2020). Evaluating circadian dysfunction in mouse models of Alzheimer’s disease: where do we stand? Front. Neurosci. 14:703. doi: 10.3389/fnins.2020.00703, PMID: 32733196 PMC7358444

[ref50] Shimizu K. Fukada Y. (2017). Stereotaxic surgery for suprachiasmatic nucleus lesions in mice. Bio. Protocol. 7:e2346. doi: 10.21769/bioprotoc.2346, PMID: 34541097 PMC8410266

[ref51] Stiedl O. Radulovic J. Lohmann R. Birkenfeld K. Palve M. Kammermeier J.. (1999). Strain and substrain differences in context- and tone-dependent fear conditioning of inbred mice. Behav. Brain Res. 104, 1–12. doi: 10.1016/s0166-4328(99)00047-9, PMID: 11125727

[ref52] Stiedl O. Tovote P. Ögren S. O. Meyer M. (2004). Behavioral and autonomic dynamics during contextual fear conditioning in mice. Auton. Neurosci. 115, 15–27. doi: 10.1016/j.autneu.2004.07.006, PMID: 15507402

[ref53] Stokes E. L. Flecknell P. A. Richardson C. A. (2009). Reported analgesic and anaesthetic administration to rodents undergoing experimental surgical procedures. Lab. Anim. 43, 149–154. doi: 10.1258/la.2008.008020, PMID: 19116297

[ref54] Talbot S. R. Biernot S. Bleich A. van Dijk R. M. Ernst L. Häger C.. (2020). Defining body-weight reduction as a humane endpoint: a critical appraisal. Lab. Anim. 54, 99–110. doi: 10.1177/0023677219883319, PMID: 31665969

[ref55] Taniguchi H. He M. Wu P. Kim S. Paik R. Sugino K.. (2011). A resource of cre driver lines for genetic targeting of GABAergic neurons in cerebral cortex. Neuron 71, 995–1013. doi: 10.1016/j.neuron.2011.07.026, PMID: 21943598 PMC3779648

[ref56] Tzeng T. C. Hasegawa Y. Iguchi R. Cheung A. Caffrey D. R. Thatcher E. J.. (2018). Inflammasome-derived cytokine IL18 suppresses amyloid-induced seizures in Alzheimer-prone mice. Proc. Natl. Acad. Sci. USA 115, 9002–9007. doi: 10.1073/pnas.1801802115, PMID: 30127003 PMC6130368

[ref57] Verhoeven K. J. F. Simonsen K. L. McIntyre L. M. (2005). Implementing false discovery rate control: increasing your power. Oikos 108, 643–647. doi: 10.1111/j.0030-1299.2005.13727.x

[ref58] Vloeberghs E. Van Dam D. Engelborghs S. Nagels G. Staufenbiel M. De Deyn P. P. (2004). Altered circadian locomotor activity in APP23 mice: a model for BPSD disturbances. Eur. J. Neurosci. 20, 2757–2766. doi: 10.1111/j.1460-9568.2004.03755.x, PMID: 15548219

[ref59] Warner E. J. Padmanabhan K. (2020). Sex differences in head-fixed voluntary running behavior in C57BL/6J mice. Eur. J. Neurosci. 51, 721–730. doi: 10.1111/ejn.14654, PMID: 31849113 PMC7069786

[ref60] Wassermann L. Helgers S. O. A. Riedesel A. K. Talbot S. R. Bleich A. Schwabe K.. (2020). Monitoring of heart rate and activity using telemetry allows grading of experimental procedures used in neuroscientific rat models. Front. Neurosci. 14:587760. doi: 10.3389/fnins.2020.587760, PMID: 33424534 PMC7793729

[ref61] Whittaker A. L. Howarth G. S. (2014). Use of spontaneous behaviour measures to assess pain in laboratory rats and mice: how are we progressing? Appl. Anim. Behav. Sci. 151, 1–12. doi: 10.1016/j.applanim.2013.11.001

[ref62] Wu M. Zhou F. Cao X. Yang J. Bai Y. Yan X.. (2018). Abnormal circadian locomotor rhythms and per gene expression in six-month-old triple transgenic mice model of Alzheimer’s disease. Neurosci. Lett. 676, 13–18. doi: 10.1016/j.neulet.2018.04.008, PMID: 29626648

[ref63] Yang F. Chen L. Yu Y. Xu T. Chen L. Yang W.. (2022). Alzheimer’s disease and epilepsy: an increasingly recognized comorbidity. Front. Aging Neurosci. 14:940515. doi: 10.3389/fnagi.2022.940515, PMID: 36438002 PMC9685172

[ref64] Zentrich E. Talbot S. R. Bleich A. Häger C. (2021). Automated home-cage monitoring during acute experimental colitis in mice. Front. Neurosci. 15:760606. doi: 10.3389/fnins.2021.760606, PMID: 34744621 PMC8570043

[ref65] Zhu S. Wang J. Zhang Y. He J. Kong J. Wang J. F.. (2017). The role of neuroinflammation and amyloid in cognitive impairment in an APP/PS1 transgenic mouse model of Alzheimer’s disease. CNS Neurosci. Ther. 23, 310–320. doi: 10.1111/cns.12677, PMID: 28191738 PMC6492725

[ref66] Zintzsch A. Noe E. Reißmann M. Ullmann K. Krämer S. Jerchow B.. (2017). Guidelines on severity assessment and classification of genetically altered mouse and rat lines. Lab. Anim. 51, 573–582. doi: 10.1177/0023677217718863, PMID: 28696160

