# Supplementary material for: Continuous locomotor activity monitoring to assess animal welfare following intracranial surgery in mice
Source: Front Behav Neurosci. 2024 Sep 4;18:1457894. doi: 10.3389/fnbeh.2024.1457894 (PMC11408287; doi:10.3389/fnbeh.2024.1457894)
Supplement: Supplementary file 1 [file Data_Sheet_1.pdf]

Supplementary Information

Continuous Locomotor Activity Monitoring to Assess Animal Welfare Following Intracranial Surgery in Mice

Mazyar Abdollahi Nejat, Oliver Stiedl*, August B. Smit, Ronald. E. van Kesteren*

*** Correspondence:**
Oliver Stiedl (oliver.stiedl@vu.nl)/ Ronald E. van Kesteren (ronald.van.kesteren@vu.nl)

1 Supplementary Methods

Stereotaxic Surgeries

All mice in this study received intracranial injections of adeno-associated viruses (AAV) for the distinct aim of a separate research study. Prior to surgery, mice were anesthetized and mounted onto a stereotaxic frame (KOPF, model 942). In order to label and/or manipulate somatostatin-positive (SST) interneurons and label parvalbumin-positive (PV) interneurons, the following viruses were injected: a 1:1 mixture of S5E2-GFP-fGP (10^{11} vc/ml; gift from Jordane Dimidschstein (Addgene #135631) (Vormstein-Schneider et al., 2020) and either floxed mCherry (hSyn-DIO-mCherry; 10^{11} vc/ml; University of North Carolina Vector Core, Chapel Hill, NC) or floxed hM4Di-mCherry (hSyn-DIO-hM4Di-mCherry; 10^9 vc/ml; University of North Carolina Vector Core, Chapel Hill, NC), all packaged as serotype 5 virus. Since sham controls were not required for this experimental design, they were not included. A total volume of 0.5 µL was infused bilaterally into the CA1 region of the hippocampus (AP: -1.8 mm; DV: -1.6 mm; ML: ±1.25), using a thin glass needle connected by Tygon tubing to a 10 µL Hamilton needle syringe, at a flow rate of 0.1 µL/min. The needle was retracted 5 min following the virus infusion to prevent backflow of the solution. After surgery, mice were placed on a heating pad in their home cage for approximately 30 min. Subsequently, mice were placed back into the DVC®-rack with their cage positioned in its original location prior to surgery.

2 Supplementary Figures

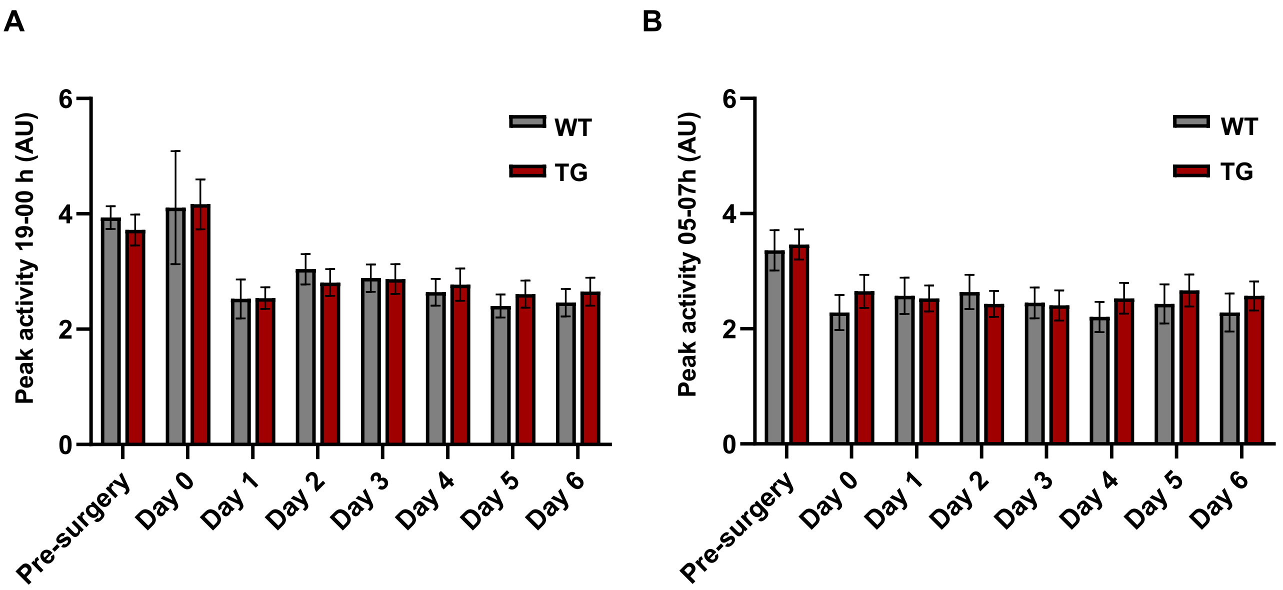

Supplementary Figure 1. Comparison between APP/PS1 TG mice and WT mice in (**A**) peak activity between 19-00 h and (**B**) peak activity between 05-07 h.

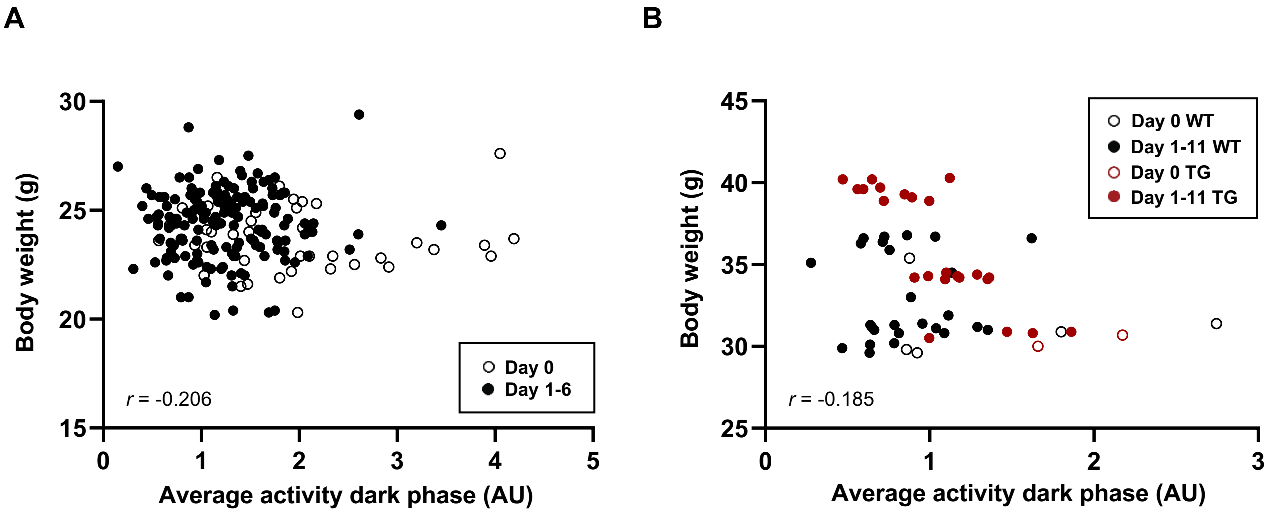

Supplementary Figure 2. Correlation analysis between post-surgery body weight and average total activity during the dark phase in (**A**) 7-8-week-old (Pearson correlation; r = -0.206, n = 196) and (**B**) 19-21-week-old mice (Pearson correlation; r = -0.185, n = 56). Pearson's r was computed for each mouse individually, and the average r value was then calculated across all mice per group.

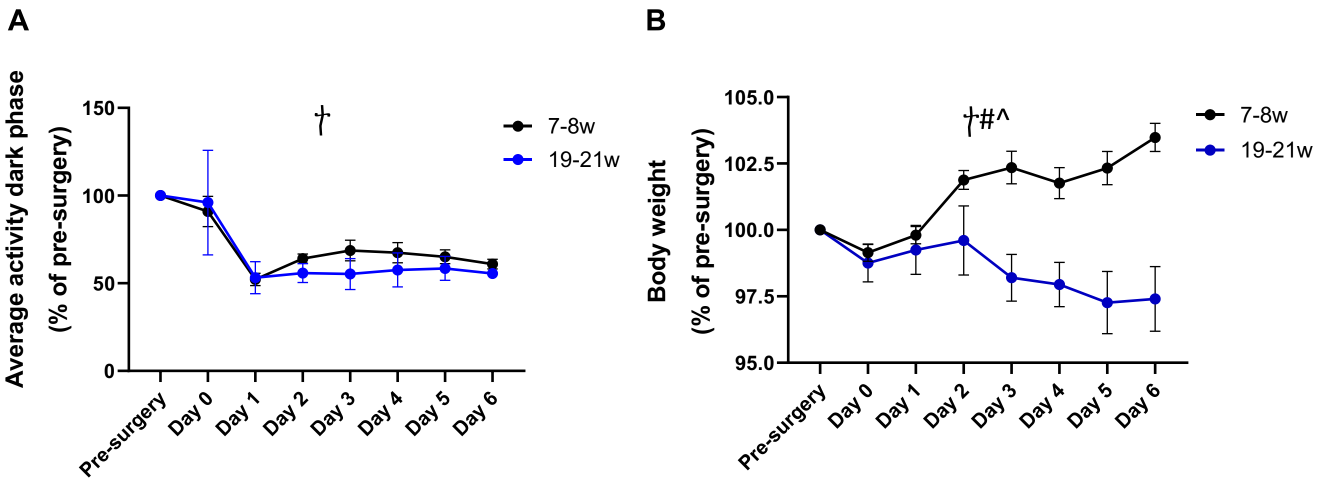

Supplementary Figure 3. Comparison between 7-8-week-old mice and 19-21-week-old mice in (**A**) average activity during the dark phase and (**B**) change in body weight. †main effect of surgery; #main effect of genotype; ^interaction effect.

3 Supplementary Tables

Supplementary Table 1. ANOVA table for total activity during the dark phase.

	F(DFn, DFd)	*p* value
Surgery	$F(1.843, 77.42) = 10.11$	$p = 0.0002$
Age	$F(1, 42) = 0.2385$	$p = 0.6279$
Surgery x Age	$F(7, 294) = 0.3134$	$p = 0.9477$

Supplementary Table 2. ANOVA table for change in body weight.

	F(DFn, DFd)	*p* value
Surgery	$F(3.946, 127.4) = 6.277$	$p = 0.0001$
Age	$F(1, 46) = 10.06$	$p = 0.0027$
Surgery x Age	$F(7, 266) = 5.694$	$p < 0.0001$

4 Supplementary Results

Circadian Locomotor Rhythm of 19-21-Week-Old Mice

Additionally, we performed mixed-effects analysis by excluding day 0 activity and body weight due to clear buprenorphine-mediated hyperactivity on day 0. The effect of surgery on total activity in WT mice did not change ($p = 0.09$ compared to $p = 0.13$ including day 0). However, excluding day 0 activity, resulted in a significant effect of surgery on total activity in 19-21-week-old TG mice ($p = 0.016$ compared to $p = 0.10$ including day 0), with Bonferroni's *post-hoc* multiple comparisons showing significantly ($p < 0.02$) lower activity on days 1, 5 and 8 compared to pre-surgery activity). Not unexpected, excluding day 0 body weights resulted in only minor statistical changes in both WT ($p = 0.71$ compared to $p = 0.84$ including day 0) and TG mice ($p = 0.40$ compared to $p = 0.38$ including day 0).
